# Reading comprehension self-efficacy and mathematical problem-solving in primary education: a serial mediation model through mathematical reasoning and critical thinking

**DOI:** 10.3389/fpsyg.2026.1841685

**Published:** 2026-07-07

**Authors:** Tunahan Filiz, Kadir Kaplan

**Affiliations:** Department of Educational Science, Bayburt University, Bayburt, Türkiye

**Keywords:** critical thinking, mathematical problem solving, mathematical reasoning, mediation analysis, primary school students, reading comprehension self-efficacy

## Abstract

**Introduction:**

Mathematical problem-solving is fundamental for academic success and real-life challenges and involves more than procedural and computational skills. As most primary school mathematics problems are text-based, reading comprehension self-efficacy may be closely associated with students’ performance in mathematical problem-solving. Grounded in Albert Bandura’s social cognitive theory, this study examined the associations between reading comprehension self-efficacy and mathematical problem-solving, focusing on the sequential indirect associations involving of mathematical reasoning and critical thinking.

**Methods:**

A quantitative, correlational research design was employed. The sample consisted of 518 fourth-grade students from 14 provinces across seven regions of Türkiye. Data were collected using validated scales measuring reading comprehension self-efficacy, critical thinking, mathematical reasoning, and mathematical problem-solving skills. Data were analyzed using correlation analysis and regression-based mediation analysis.

**Results:**

The findings revealed that mathematical reasoning and critical thinking partially accounted for the association between reading comprehension self-efficacy and mathematical problem-solving. The findings indicated significant indirect effects through critical thinking skills (*β* = 0.18, *K*^2^ = 0.23), mathematical reasoning skills (*β* = 0.04, *K*^2^ = 0.19), and the sequential pathway from mathematical reasoning to critical thinking (*β* = 0.01, *K*^2^ = 0.03). Although the sequential mediation effect was statistically significant, its effect size was relatively small compared with the individual mediation effects, particularly that of critical thinking skills.

**Discussion:**

These results highlight the interconnected roles of foundational cognitive processes in relation to higher-order mathematical problem-solving skills. The study contributes to the literature by emphasizing the relevance of considering reading comprehension self-efficacy, mathematical reasoning, and critical thinking as interconnected factors in early mathematics education.

## Introduction

Mathematical problem-solving skills (MPSS) is a core skill in basic education, vital for academic success and tackling real-world challenges (NCTM, 2000). However, effective problem-solving involves more than procedural knowledge or numerical computation. It depends on a dynamic interplay of cognitive, metacognitive, and affective factors that shape student performance (Schoenfeld, 2009; Verschaffel et al., 2000). Among these, reading comprehension plays a significant role. Since most primary mathematics problems are presented verbally, students must decode linguistic information, identify relevant mathematical content, and construct appropriate mental representations before applying computational procedures (Pape, 2004). In the present study, reading comprehension self-efficacy was used because mathematical word problems require students to understand and interpret written information before applying mathematical procedures. RCSE refers to students’ beliefs about their ability to comprehend written texts, identify relevant information, and construct meaning from problem statements. This construct differs from traditional mathematics self-efficacy, which refers to students’ confidence in performing mathematical tasks, applying procedures, or solving mathematics problems. Therefore, RCSE was not treated as a substitute for mathematics self-efficacy but as a language-related self-belief that may be associated with the comprehension and representation demands of mathematical word problem-solving.

According to Bandura’s (1997) social cognitive theory, individuals’ beliefs in their own self-efficacy play a crucial role in shaping their motivation, persistence, and overall performance in accomplishing tasks. Students with higher reading comprehension self-efficacy (RCSE) approach math problems more confidently and strategically, enhancing their performance. This relationship, however, may be mediated by higher-order cognitive processes that are theorized to link text comprehension to mathematical reasoning skills. Critical thinking skills and mathematical reasoning skills are two such essential processes. Mathematical reasoning involves recognizing patterns, drawing logical conclusions, and justifying solutions (Lithner, 2008). Similarly, critical thinking enables students to evaluate information and arguments, identify problem requirements, and monitor progress (Lipman, 2003). Although mathematical reasoning and critical thinking are closely related cognitive processes, they were treated as operationally distinct constructs in this study. Mathematical reasoning refers primarily to students’ ability to identify mathematical relationships, recognize patterns, construct logical connections, and represent problem situations in mathematical terms. In contrast, critical thinking refers to students’ ability to evaluate information, question the appropriateness of solution strategies, compare alternatives, and monitor the adequacy of their reasoning. Thus, mathematical reasoning was conceptualized as a more domain-specific process for constructing mathematical meaning, whereas critical thinking was conceptualized as a broader evaluative process for judging and regulating problem-solving decisions. This distinction is particularly important in mathematical word problems, where students first need to make sense of the mathematical relationships embedded in the text and then evaluate whether a selected solution strategy is appropriate. Nevertheless, given fourth-grade students’ developmental level, the boundaries between these two constructs may be permeable. Therefore, the sequential ordering tested in this study should be interpreted as a theoretically informed statistical model rather than as evidence of sharply separated or developmentally fixed cognitive stages.

The relationship between RCSE and MPS may thus be mediated by reasoning and critical thinking. In Pólya’s (1945) problem-solving model, understanding the problem and devising a plan require comprehension and reasoning. Schoenfeld’s (1992) framework further emphasizes the integration of metacognitive processes, beliefs, and cognitive resources. From this perspective, students’ reading self-efficacy may be associated with their willingness to engage with problem texts, while reasoning and critical thinking are theorized to support the transformation of textual information into effective mathematical strategies.

## Theoretical framework

This study integrates three theoretical perspectives—Bandura’s Social Cognitive Theory, cognitive-developmental theories of mathematical thinking, and modern models of MPS—forming a comprehensive framework to examine how cognitive processes and self-efficacy beliefs affect mathematical performance.

### The association between reading comprehension self-efficacy and mathematical problem-solving skills

Theoretically, the relationship between MPSS and RCSE is based on several convergent viewpoints that shed light on the direct and indirect ways that attitudes about reading affect mathematical results. Early failures can reduce self-efficacy and hinder reading and mathematical reasoning (Stanovich, 1986). Mathematical word problems require linguistic and mathematical processing (Daroczy et al., 2015). According to Kintsch and Greeno (1985), solving such problems involves building a textbase, situation, and problem model, each of which demands advanced reading comprehension. RCSE has been found to be associated with MPSS (Vilenius-Tuohimaa et al., 2008; Pimperton and Nation, 2010; Fuchs et al., 2015), and beliefs about reading ability play a crucial motivational role in this context. Low self-efficacy increases anxiety, consuming working memory resources needed for problem representation (Bandura, 1997; Ashcraft and Kirk, 2001). The psychological component of students’ ideas about their RCSE is just as important as their reading comprehension ability.

### The association between reading comprehension self-efficacy and critical thinking skills (CTS)

Reading enables exposure to diverse perspectives and reasoning. Students with higher RCSE may be more willing to engage with written material, and such engagement has been discussed in relation to critical thinking (Cunningham and Stanovich, 1998). Although self-efficacy and critical thinking predict academic success, their interaction explains how affective beliefs support higher-order cognition. CTS requires effortful reasoning and metacognitive control (Stanovich and West, 2000; Kahneman, 2011). Students with higher RCSE may report greater confidence in engaging with text-based tasks, which may be associated with deeper processing, argument evaluation, and comprehension monitoring (Schunk and Zimmerman, 2007; Facione, 1990; Schraw and Dennison, 1994). Self-efficacy promotes epistemic curiosity and open-mindedness (Litman, 2008; Ku and Ho, 2010). Within the self-regulated learning framework, students with high RCSE set deeper comprehension goals and apply critical analysis (Zimmerman, 2002; Schunk, 1991). Research indicates that CTS facilitates the connection between self-efficacy and problem-solving, encompassing RCSE (Hotaman, 2008; Taşgın and Dilek, 2023; Wang et al., 2024; Exintaris et al., 2023).

### The association between reading comprehension self-efficacy and mathematical reasoning skills (MRS)

Although analyzing patterns, constructing logical arguments, and formulating generalizations are all essential components of MRS, students’ self-efficacy beliefs significantly impact how well this cognitive skill is activated and used (Vale et al., 2017). RCSE has also been found to be associated with MRS, which depends on constructing logical relations and schemas from linguistic input (Boonen et al., 2016; Fuchs et al., 2015). Understanding mathematical problems involves identifying relationships expressed linguistically (Nathan et al., 1992; Daroczy et al., 2015). Students who are confident in reading attend to these cues and build accurate representations. RCSE encourages engagement with complex problems and supports schema development, enhancing analogical reasoning (Gentner and Smith, 2013). Since word problems are linguistic, RCSE appears to be associated with MRS, potentially supporting persistence and comprehension throughout problem solving (Krawitz and Schukajlow, 2018; Kim, 2021; Verschaffel et al., 2000).

### The relationship of reading comprehension self-efficacy with mathematical problem-solving skills and critical thinking skills, and mathematical reasoning skills

Empirical evidence shows reciprocal interactions among RCSE, MRS, CTS, and MPSS (Landa et al., 2025; Schöber et al., 2018). RCSE has been associated with MRS and CTS, which in turn relate to problem-solving performance. Word problems link linguistic and mathematical domains (Daroczy et al., 2015). Cognitive load and working memory theories (Sweller, 1988; Baddeley, 2003) explain how limited resources are shared among comprehension, reasoning, and evaluation processes. The theoretical distinction between MRS and CTS in the present model is further supported by the operational indicators of mathematical reasoning in problem-solving contexts. Mathematical reasoning has been described through indicators such as representing mathematical situations verbally, visually, or diagrammatically, identifying known and unknown information, making assumptions, organizing information through mathematical manipulation, constructing justifications, drawing conclusions from mathematical expressions, checking results, and making generalizations in relation to problem-solving stages (Özdemir, 2023; Pólya, 1973; Lithner, 2000; Herawati and Amelia, 2020). These indicators show that MRS is primarily concerned with constructing and justifying mathematical meaning within the problem situation. CTS, by contrast, refers to broader evaluative and metacognitive processes, such as examining assumptions, monitoring the consistency of reasoning, evaluating the appropriateness of selected strategies, and judging the plausibility of solutions (Facione, 1990; Schraw and Dennison, 1994; Ku and Ho, 2010). Therefore, although MRS and CTS may overlap during fourth-grade students’ problem-solving processes, they are theoretically differentiated in this study as mathematics-specific constructive reasoning and broader evaluative-regulatory thinking, respectively. RCSE supports accurate problem understanding and strategy formation (Vilenius-Tuohimaa et al., 2008). Studies indicate that RCSE is significantly associated with problem-solving and reasoning (Erdem, 2016; Ural and Ülper, 2013) and that critical thinking is a key determinant of mathematical success (Can, 2020; Landa et al., 2025; Ocak et al., 2023; Sternberg and Hayes, 2025; Türnüklü and Yeşildere, 2005). This comprehensive theoretical framework has articulated an integrated Cognitive-Affective Mediation Model, positioning RCSE as a foundational affective variable that may be associated with MPSS through theorized mediating pathways involving CTS and MRS. The model recognizes the domain-integrative nature of word problem-solving, synthesizes many theoretical viewpoints, offers testable empirical predictions, and acknowledges reciprocal developmental dynamics while concentrating analytically on specific directed paths. Each model’s dyadic relationship—self-efficacy to reasoning, self-efficacy to critical thinking, reasoning to problem-solving, critical thinking to problem-solving, and reasoning to critical thinking—has strong theoretical underpinnings derived from well-established psychological and educational theories, as shown by the theoretical analyses in this study.

### This study

**Figure 1:**
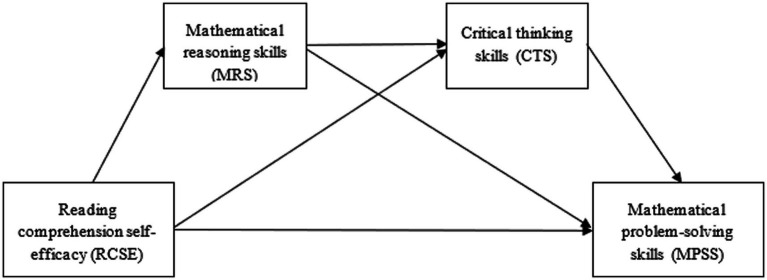
Testing the serial mediation model.

The connection between MPSS and RCSE is particularly salient in primary school, where both skills develop (Fuchs et al., 2008). Younger children’s self-efficacy is more malleable (Schunk and Meece, 2006), and reading comprehension underpins mathematical reasoning in word problems (NCTM, 2000). The primary school years are crucial for forming these intertwined skills (Jordan et al., 2009). Therefore, this study examines the associations between RCSE and MPSS, focusing on the sequential mediating roles of MRS and CTS among primary school students. Although cross-sectional, it captures concurrent relationships within this developmental system the study aims to capture concurrent relationships within this developmental system and extends previous findings (Vilenius-Tuohimaa et al., 2008; Ku and Ho, 2010) by integrating them into a unified theoretical model. To validate the model based on the study’s theoretical framework, the following hypotheses were tested (Figure 1):

*H1*: There is a significant association among primary school students' RCSE, mathematical reasoning skills, critical thinking skills, and MPSS.

*H2*: RCSE is significantly associated with MPSS among primary school students.

*H3*: Critical thinking skills play a significant mediating role in the association between RCSE and MPSS.

*H4:* Mathematical reasoning skills play a significant mediating role in the association between RCSE and MPSS.

*H5*: Mathematical reasoning skills and critical thinking skills serve as sequential mediators in the association between RCSE and MPSS.

## Method

### Research model

The study is a quantitative study based on a correlational research model. According to Fraenkel et al. (2015), this model allows for determining the presence, direction, and strength of relationships among variables. Within this framework, confirmatory factor analysis (CFA) was conducted using LISREL to evaluate the measurement model. Subsequently, regression-based mediation and serial mediation analyses were performed using the PROCESS macro for SPSS. In this study, the relationships among RCSE, MPSS, critical thinking, and mathematical reasoning were investigated. Moreover, given the focus on the mediating roles of critical thinking and mathematical reasoning in MPSS, a regression-based mediation approach was employed to examine the indirect relationships among these variables within the proposed theoretical framework.

### Participants and procedures

A stratified sampling method was employed to identify and select the participants for the study. This method was chosen because it has a high power to represent the population and allows for generalizations. First, 14 provinces were randomly selected to represent each of Turkey’s seven geographical regions. Subsequently, three official primary schools from these provinces were selected for the sample through a random sampling procedure. Participation was voluntary, and data were collected during individual classroom visits. Prior to data collection, written informed consent was obtained from the parents or legal guardians of the students, and assent was obtained from the participating children. This study was conducted in accordance with the principles of the Declaration of Helsinki. Ethical approval was obtained from the Social and Human Sciences Ethics Committee of Bayburt University (Approval Date: 12 March 2025; Protocol No.: E-79126184-050.99-262846). The data collection sessions were scheduled in coordination with school administrators and teachers at convenient times, and the entire procedure was finalized within 2 days. Data were collected from 518 fourth-grade primary school students. Fourth-grade students were selected because this grade level represents a critical developmental stage at which students are expected to integrate reading comprehension, reasoning, and problem-solving skills within the mathematics curriculum. In the Turkish primary school curriculum, mathematical problem-solving tasks become increasingly complex in Grade 4 and require students to interpret, analyze, and solve multi-step word problems. Furthermore, from a developmental perspective, students at this age are approaching the transition from concrete operational thinking toward more advanced forms of reasoning, making Grade 4 particularly appropriate for examining the relationships among reading comprehension self-efficacy, mathematical reasoning, critical thinking, and mathematical problem-solving skills. All participants were Turkish citizens. The gender distribution of participants was 265 (51.16%) girls and 253 (48.84%) boys.

## Instruments

### Reading comprehension self-efficacy scale

The RCSE scale is a measurement tool created to determine primary school students’ self-efficacy perceptions regarding reading comprehension (Kula and Budak, 2020). The scale comprises 29 items and demonstrates a unidimensional structure. Developed as a three-point Likert scale with total scores ranging from 29 to 87, the instrument’s construct validity was confirmed through exploratory and confirmatory factor analyses. Findings from the EFA demonstrated that the scale’s unidimensional structure explained 30.36% of the total variance, with the first and second factors having eigenvalues of 9.109 and 1.360, respectively. All items in the scale had factor loadings above 0.30. The fit indices obtained from CFA (confirmatory factor analysis) indicate that the model is acceptable (*χ*^2^/df = 2.12; NFI = 0.95; NNFI = 0.97; RFI = 0.95; IFI = 0.98; CFI = 0.98; RMSEA = 0.04). The Cronbach’s alpha coefficient for the entire scale was found to be 0.92, indicating a high level of internal consistency. In addition, the reliability of the scale was tested using the Guttman Split-Half correlation method. The correlation coefficient was found to be 0.83. The reliability analysis conducted as part of this study revealed that the Cronbach’s alpha coefficient for the general scale was 0.91, demonstrating a high level of internal consistency. The CFA conducted on the data obtained in the present study indicated an acceptable model fit for the RCSE scale (*χ*^2^/df = 1.988, CFI = 0.909, TLI = 0.900, RMSEA = 0.044, 90% CI [0.039, 0.048], SRMR = 0.043). According to the criteria proposed by Hu and Bentler (1999), the RMSEA and SRMR values indicated good model fit, whereas the CFI and TLI values reflected acceptable fit.

### Critical thinking scale

The critical thinking scale is a tool developed to measure students’ critical thinking dispositions (Uluçinar and Akar, 2021). The scale consists of 18 items and has four factors: “Curiosity,” “Skepticism,” “Open-mindedness,” and “Biasness.” The factor loadings of the scale items range from 0.35 to 0.74. The total score obtainable from the scale ranges from a minimum of 18 to a maximum of 72. The construct validity of the scale was assessed through CFA, and the resulting fit indices indicated an excellent and acceptable model fit (*χ*^2^/df = 1.33; RMR = 0.047; RMSEA = 0.03; IFI = 0.95; CFI = 0.95; TLI = 0.94). Cronbach’s alpha coefficient for the entire scale was calculated as 0.74. Cronbach’s alpha coefficients for the subscales were determined as follows: Skepticism (0.67), Curiosity (0.60), Open-mindedness (0.62), and Biasness (0.61). These results confirm that the scale is both a valid and reliable instrument. The reliability analysis performed in this study yielded a Cronbach’s alpha coefficient of 0.75 for the general scale, demonstrating an acceptable level of internal consistency. The CFA conducted on the present sample (*N* = 518) indicated an acceptable model fit for the CTS scale (*χ*^2^/df = 2.206, CFI = 0.936, TLI = 0.924, RMSEA = 0.048, 90% CI [0.041, 0.056], SRMR = 0.045). According to the criteria proposed by Hu and Bentler (1999), the RMSEA and SRMR values indicated good model fit, whereas the CFI and TLI values reflected acceptable fit.

### Mathematical reasoning scale

The mathematical reasoning test is an assessment tool developed to measure the MRS of year 4 primary school students (Gürbüz and Erdem, 2016). The assessment tool comprises 14 items, including both multiple-choice and open-ended questions. Its content validity and suitability for fourth-grade primary school students were confirmed through expert evaluations conducted by two mathematics educators and four mathematics teachers. The test is administered over a period of 40 min. The test, adapted into Turkish by the researchers based on the graded scoring rubric developed by Reys et al. (1995), yields scores ranging from 0 to 70, with higher scores reflecting stronger performance. The scale’s reliability was determined to be 0.86 using Cronbach’s alpha. The assessment criteria for the test are classified according to the accuracy of the answers given and the level of justification: “Correct justification” (5 points), “Incorrect answer – correct justification” (4 points), “Partially correct justification” (3 points), “Incorrect answer – partially correct justification” (2 points), “Incorrect justification” (1 point), “Incorrect answer – incorrect justification” (0 points), and “No justification” (0–1 points). The reliability analysis showed that the measurement instrument demonstrated strong internal consistency, with a Cronbach’s alpha coefficient of 0.86. Unlike the other instruments used in this study, the Mathematical Reasoning Scale (MRS) is a performance-based assessment consisting of multiple-choice and open-ended items evaluated using an analytic scoring rubric. Therefore, construct validity evidence for this instrument was established through expert review, scoring procedures, and reliability analyses reported in the original development study rather than through a latent-variable CFA framework.

### Mathematical problem-solving scale

The MPS scale is a tool developed to measure primary school students’ MPSS (Değirmenci and Deringöl, 2024). The scale is composed of 16 items, which are distributed across three underlying factors: “Self-Assessment in Problem Solving,” “Problem Solving Knowledge,” and “Metacognition in Problem Solving.” All items are rated on a five-point Likert scale, with factor loadings ranging from 0.39 to 0.75; the scale contains no reverse-coded items, and total scores range from 16 to 80. The scale’s construct validity was examined using CFA, and its three-factor structure was confirmed. The fit indices obtained were found to be acceptable (*χ*^2^/df = 2.225; RMR = 0.077; CFI = 0.923; RMSEA = 0.080; GFI = 0.913; AGFI = 0.914). The Cronbach’s alpha coefficients for the subscales of the instrument were calculated as 0.85, 0.84, and 0.75, respectively. The reliability analysis for the overall scale produced a Cronbach’s alpha of 0.86 and a Guttman Split-Half coefficient of 0.81, indicating strong internal reliability. In this study, the reliability analysis revealed a Cronbach’s alpha coefficient of 0.87 for the overall scale, demonstrating a high degree of internal consistency. The CFA conducted on the present sample (*N* = 518) indicated an acceptable model fit for the MPSS scale (*χ*^2^/df = 2.087, CFI = 0.946, TLI = 0.936, RMSEA = 0.046, 90% CI [0.037, 0.055], SRMR = 0.051). According to the criteria proposed by Hu and Bentler (1999), the RMSEA and SRMR values indicated good model fit, whereas the CFI and TLI values reflected acceptable fit.

### Data analysis

Data analysis was conducted using IBM SPSS Statistics 25, the PROCESS macro, and LISREL for CFA. First, missing data and outliers were examined. Eight cases with incomplete responses were removed from the original dataset of 530 observations. Prior to the deletion procedure, Little’s MCAR test was conducted to examine the missing-data mechanism. The results indicated that the missing values were missing completely at random, *χ*^2^(640) = 42.41, *p* = 1.000. Therefore, listwise deletion was considered an appropriate method for handling the small amount of missing data. Multivariate outliers were identified using Mahalanobis distance coefficients. Consistent with the recommendation of Tabachnick and Fidell (2013), cases with Mahalanobis distance values exceeding the critical chi-square value at *p* < 0.001 [*χ*^2^(4) = 18.47] were classified as multivariate outliers. Based on this criterion, four cases (S237, S250, S364, and S391) were identified as multivariate outliers and excluded from subsequent analyses. A sensitivity analysis was conducted by re-running the primary analyses with the four excluded cases included in the dataset. The pattern, direction, and statistical significance of the results remained unchanged, indicating that the findings were robust to the exclusion of these multivariate outliers. Consequently, the final analyses were conducted on 518 observations. Subsequently, the assumption of normality was assessed using skewness and kurtosis values, which were found to be within the acceptable range of −1 to +1 (Tabachnick and Fidell, 2013).

Descriptive statistics were then reported, followed by Pearson correlation analysis to examine the relationships among RCSE, MPSS, critical thinking skills, and mathematical reasoning skills. Prior to the main analyses, the validity and reliability of the measurement instruments were evaluated. Internal consistency was assessed using Cronbach’s alpha coefficients. In addition, CFA was conducted using LISREL to examine the construct validity of the scales. Model fit was evaluated using multiple fit indices, including *χ*^2^/df, SRMR, CFI, TLI, and RMSEA. Following Hu and Bentler (1999), CFI and TLI values of 0.95 or higher, RMSEA values of 0.06 or lower, and SRMR values of 0.08 or lower were considered indicative of good model fit, whereas CFI and TLI values of 0.90 or higher, RMSEA values of 0.08 or lower, and SRMR values of 0.10 or lower were considered indicative of acceptable model fit.

Because several constructs in the study were assessed using self-report instruments, potential common method variance (CMV) was also examined. However, the study did not rely exclusively on self-report data, as mathematical reasoning skills were measured through an objective performance-based cognitive assessment scored using an analytic rubric. To evaluate the possible influence of CMV among the self-report measures, Harman’s single-factor test and CFA-based procedures were conducted using only the self-report items. Specifically, the proportion of variance explained by a single factor was examined, and the fit of a one-factor measurement model was compared with that of the theoretically specified measurement model.

To address concerns regarding measurement error associated with observed composite scores, an additional latent variable Structural Equation Modeling (SEM) analysis corresponding to the hypothesized serial mediation framework was conducted as a robustness check. The latent SEM modeled reading comprehension self-efficacy, mathematical reasoning, critical thinking, and mathematical problem-solving skills as latent constructs and estimated the direct, indirect, and serial indirect effects within a single structural model. The substantive conclusions obtained from the latent SEM were generally consistent with those obtained from the PROCESS analysis. Detailed latent SEM results are reported in the Appendix A.

Although CFA was conducted within a latent-variable framework, the mediation analyses were performed using observed composite scores through Hayes’ PROCESS macro. PROCESS was preferred because it provides a flexible regression-based approach for estimating direct, indirect, and serial indirect effects using bootstrap confidence intervals, which are widely recommended for mediation analysis. However, because PROCESS relies on observed variables, measurement error is not modeled explicitly, and the resulting path coefficients may therefore represent conservative estimates of the associations among the study variables (Hayes and Preacher, 2014; Judd et al., 2014). Finally, the hypothesized mediation and serial mediation relationships were tested using Hayes’ (2018) PROCESS macro (Model 6) based on a regression framework. The significance of indirect effects was assessed using a bootstrap procedure with 5,000 bias-corrected resamples and a 95% confidence interval. Indirect effects were considered statistically significant when the confidence intervals did not include zero.

## Results

### The association among RCSE, CTS, MRS and MPSS

Descriptive statistics and Pearson correlation coefficients among the study variables are presented in Table 1. Within the scope of the study’s first hypothesis (H1), the relationships between RCSE, critical thinking, mathematical reasoning, and MPSS were examined, and the results are presented in Table 1.

**Table 1:** Pearson correlations among study variables.

Variable	1	2	3	4
1. Reading comprehension self-efficacy (RCSE)	1			
2. Critical thinking skills (CTS)	0.55^**^	1		
3. Mathematical reasoning skills (MRS)	0.33^**^	0.26^**^	1	
4. Mathematical problem-solving skills (MPSS)	0.51^**^	0.50^**^	0.27^**^	1
Mean	71.95	51.97	31.57	63.66
SD	10.40	8.06	16.56	10.62
*a*	0.91	0.75	0.86	0.87

^*^p < 0.05, ^**^p < 0.01.

As shown in Table 1, significant positive correlations were observed among all study variables. RCSE was positively correlated with CTS (*r* = 0.55, *p* < 0.01), MRS (*r* = 0.33, *p* < 0.01), and MPSS (*r* = 0.51, *p* < 0.01). CTS was also positively associated with MRS (*r* = 0.26, *p* < 0.01) and MPSS (*r* = 0.50, *p* < 0.01). In addition, a positive and significant correlation was found between MRS and MPSS (*r* = 0.27, *p* < 0.01). Overall, these findings indicate that higher levels of reading comprehension self-efficacy, critical thinking skills, mathematical reasoning skills, and mathematical problem-solving skills are associated with one another, thereby supporting the first hypothesis (H1).

### Common method variance diagnostics

As an additional validity check, common method variance diagnostics were performed using both Harman’s single-factor test and confirmatory factor analysis procedures. The findings are summarized in Table 2.

**Table 2:** Common method variance diagnostics.

Analysis	Result
Harman’s single-factor test	Single factor explained 20.78% of total variance
One-factor CFA model	*χ*^2^(1890) = 4207.21, CFI = 0.847, TLI = 0.842, RMSEA = 0.049, SRMR = 0.085
Theoretical measurement model	*χ*^2^(1880) = 2470.26, CFI = 0.961, TLI = 0.960, RMSEA = 0.025, SRMR = 0.057

To assess common method variance, Harman’s single-factor test was first conducted. The results indicated that a single factor accounted for 20.78% of the total variance, which is substantially below the commonly accepted threshold of 50%. This finding suggests that no dominant common method factor was present in the data.

Additionally, a one-factor confirmatory factor analysis model, in which all self-report items loaded onto a single latent factor, was estimated and compared with the theoretically specified measurement model. The one-factor model demonstrated substantially poorer fit (CFI = 0.847, TLI = 0.842, RMSEA = 0.049, SRMR = 0.085) than the theoretical measurement model (CFI = 0.961, TLI = 0.960, RMSEA = 0.025, SRMR = 0.057). Taken together, these findings suggest that common method variance is unlikely to represent a serious threat to the validity of the reported relationships among the study variables.

### Assumption checks for regression-based analyses

Prior to the mediation analyses, the assumptions required for regression-based analyses were examined. The assumption of normality was assessed using skewness and kurtosis values, which ranged between −1 and +1, indicating that the data were approximately normally distributed (Tabachnick and Fidell, 2013). In addition, multicollinearity diagnostics were conducted to ensure that the independent and mediating variables were not highly correlated. The obtained VIF (1.14–1.51), Tolerance (0.66–0.88), and Condition Index (4.99–20.26) values satisfied the criteria of VIF < 10, CI < 30, and Tolerance > 0.20, indicating that multicollinearity was not a concern (Tabachnick and Fidell, 2013).

### The mediating role of CTS in the association between RCSE and MPSS

**Figure 2:**
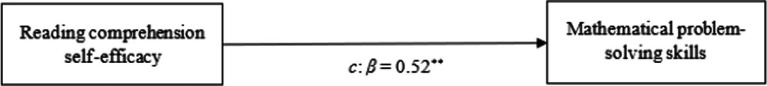
The overall effect of RCSE on MPSS. *p < 0.05, **p < 0.01.

Within the scope of the study’s second hypothesis (H2), the association between RCSE and MPSS was examined, and the findings are presented in Figure 2.

Based on Figure 2, students’ RCSE was significantly associated with their MPSS (path c; *β* = 0.52, SE = 0.04, *p* < 0.01). The results illustrating the mediating role of CTS in the relationship between RCSE and MPSS are provided in Table 3.

**Table 3:** Regression results and indirect effects for critical thinking as a mediator.

Antecedent	Path	Consequent						
		*M* (CTS)				*Y* (MPSS)		
		*β*	SE	*p*		*β*	SE	*p*
*X* (RCSE)	*a*	0.42	0.03	<0.01	*c*’	0.34	0.04	<0.01
*M* (CTS)					*B*	0.42	0.06	<0.01
Constant	*i_m_*	21.54	2.08	<0.01	*i_y_*	17.42	2.95	<0.01
*R* ^2^		0.30				0.33		
*F* (df)		219.38^***^ (1, 516)				125.42^***^ (2, 515)		

*N* = 518. Unstandardized regression coefficients are reported. Indirect effects were estimated using 5,000 bootstrap samples (95% confidence interval). SE denotes standard error. *p < 0.05, **p < 0.01, ***p < 0.001.

**Figure 3:**
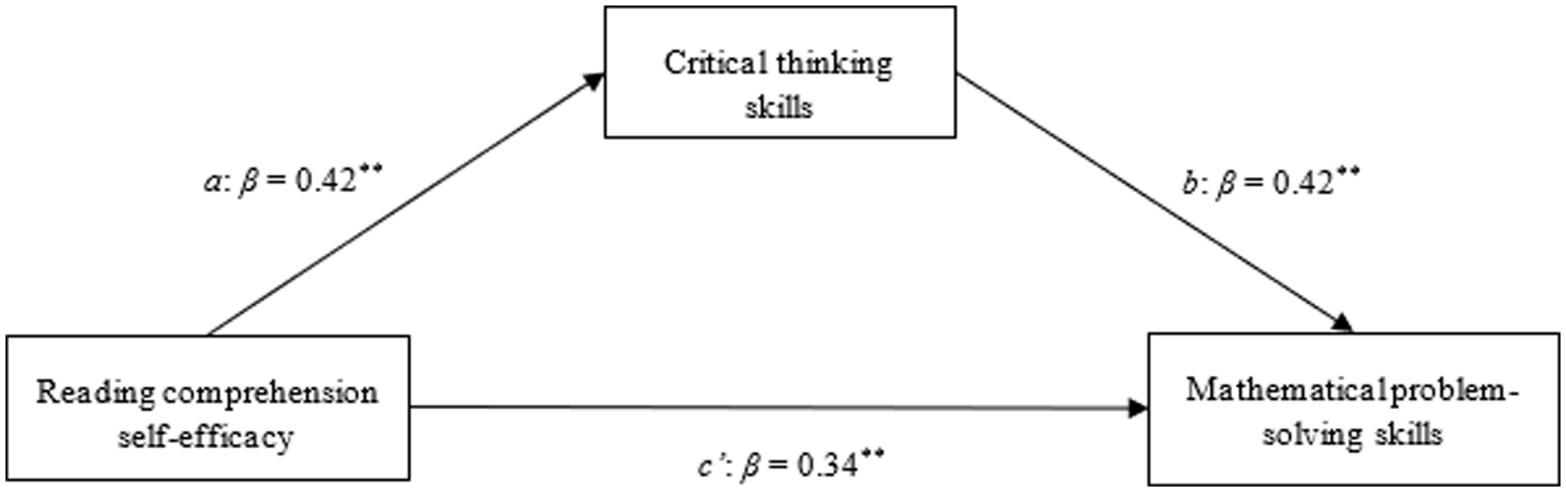
Critical thinking skills as a mediator in the association between reading comprehension self-efficacy and MPSS. *p < 0.05, **p < 0.01.

According to Table 3, RCSE was significantly associated with CTS, the proposed mediator (path *a*; *β* = 0.42, SE = 0.03, *p* < 0.01). Moreover, CTS was significantly associated with MPSS (path *b*; *β* = 0.42, SE = 0.06, *p* < 0.01). In addition, the indirect effect of RCSE on MPSS through critical thinking was statistically significant (path *ab*; *β* = 0.18; 95% CI [0.12, 0.23], SE = 0.03). When CTS, as the mediator, was included in the model, RCSE remained significantly associated with MPSS (path *c′; β* = 0.34, SE = 0.04, *p* < 0.01). In other words, even after accounting for CTS, RCSE remained a significant predictor of MPSS. This indicates that critical thinking partially mediates the association between RCSE and MPSS. Furthermore, the standardized indirect effect size of RCSE on MPSS was substantial and significant (*K*^2^ = 0.23). These findings support the third hypothesis, indicating that CTS plays a significant mediating role in the association between RCSE and MPSS (see Figure 3).

### The mediating role of MRS in the association between RCSE and MPSS

As part of the study’s fourth hypothesis (H4), Table 4 presents the results concerning the mediating role of MRS in the association between primary school students’ RCSE and MPSS.

**Table 4:** Regression results and indirect effects for MRS as a mediator.

Antecedent	Path	Consequent						
		*M* (MRS)				*Y* (MPSS)		
		*β*	SE	*p*		*β*	SE	*p*
*X* (RCSE)	*a*	0.53	0.07	<0.01	*c*’	0.48	0.04	<0.01
*M* (MRS)					*B*	0.08	0.03	<0.05
Constant	*i_M_*	−6.61	4.80	0.17	*i_y_*	26.94	2.80	<0.01
*R* ^2^		0.11				0.27		
*F* (df)		64.54^*^ (1, 516)				94.71^*^ (2, 515)		

*N* = 518. Unstandardized regression coefficients are reported. Indirect effects were estimated using 5,000 bootstrap samples (95% confidence interval). SE denotes standard error. *p < 0.05, **p < 0.01, ***p < 0.001.

**Figure 4:**
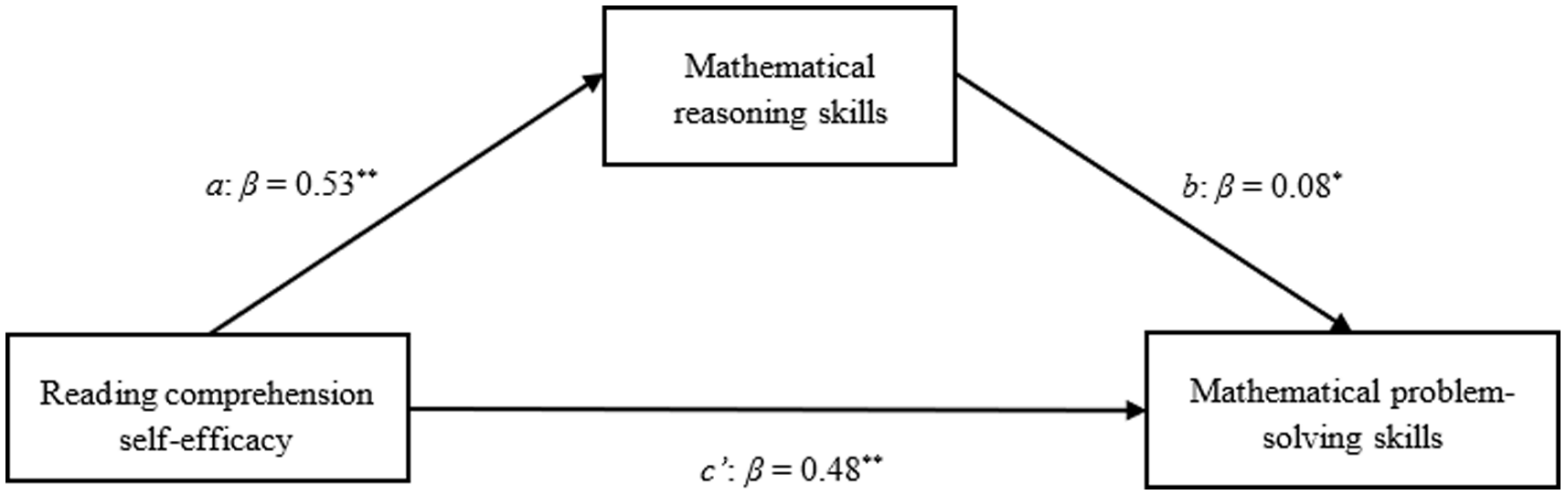
MRS as a mediator in the association between RCSE and MPSS. *p < 0.05, **p < 0.01.

According to Table 4, RCSE was significantly associated with MRS, the proposed mediator (path *a*; *β* = 0.53, SE = 0.07, *p* < 0.01). Similarly, MRS was significantly associated with MPSS (path *b*; *β* = 0.08, SE = 0.03, *p* < 0.05). Furthermore, the indirect effect of RCSE on MPSS was significant through MRS (path *ab*; *β* = 0.04; 95% CI [0.01, 0.07], SE = 0.02). When MRS was included as the mediator, RCSE remained significantly associated with MPSS (path *c′; β* = 0.48, SE = 0.04, *p* < 0.01). This indicates that RCSE remained a significant predictor of MPSS even after accounting for MRS. This finding suggests that MRS serves as a partial mediator in the association between RCSE and MPSS. Moreover, the standardized indirect effect of RCSE on MPSS was substantial and statistically significant (*K*^2^ = 0.19). These findings support the fourth hypothesis, indicating that MRS plays a significant mediating role in the association between RCSE and MPSS (see Figure 4).

### The sequential mediating roles of MRS and CTS in the association between RCSE and MPSS

In line with the fifth hypothesis (H5), Table 5 presents the findings on the sequential mediating roles of MRS and CTS in the association between primary school students’ RCSE and MPSS.

**Table 5:** Regression results and indirect effects for the sequential mediating roles of MRS and CTS.

Antecedent	Path	Consequent										
		*M_1_* (MRS)				*M_2_* (CTS)				*Y* (MPSS)		
		*β*	SE	*p*		*β*	SE	*p*		*β*	SE	*p*
*X* (RCSE)	*a_1_*	0.53	0.07	<0.01	*a_2_*	0.40	0.03	<0.01	*c*’	0.32	0.05	<0.01
*M_1_* (MRS)					*d_21_*	0.04	0.02	<0.05	*b_1_*	0.06	0.02	<0.05
*M_2_* (CTS)									*b_2_*	0.41	0.06	<0.01
Constant	*i_M1_*	−6.62	4.80	0.17	*i_M2_*	21.83	2.07	< 0.01	*i_y_*	18.10	2.95	< 0.01
*R* ^2^		0.11				0.31				0.33		
*F* (df)		64.54^*^ (1, 516)				113.32^*^ (2, 515)				86.14^*^ (3, 514)		

Antecedent	Path	Consequent										
		*M*_1_ (CTS)				*M*_2_ (MRS)				*Y* (MPSS)		
		*β*	SE	*p*		*β*	SE	*p*		*β*	SE	*p*
*X* (RCSE)	*a_1_*	0.42	0.03	<0.01	*a_2_*	0.43	0.08	<0.01	*c*’	0.32	0.05	<0.01
*M_1_* (CTS)					*d_21_*	0.24	0.10	<0.05	*b_1_*	0.41	0.06	<0.01
*M_2_* (MRS)									*b_2_*	0.06	0.02	<0.05
Constant	*i_M1_*	21.54	2.08	<0.01	*i_M2_*	−11.70	5.26	<0.05	*i_y_*	18.10	2.95	<0.01
*R* ^2^		0.30				0.12				0.33		
*F* (df)		219.38^*^ (1, 516)				35.24^*^ (2, 515)				86.14^*^ (3, 514)		

*N* = 518. Unstandardized regression coefficients are reported. The bootstrap procedure was performed with 5,000 resamples, and indirect effects were evaluated based on 95% confidence intervals. SE denotes standard error. *p < 0.05, **p < 0.01, ***p < 0.001.

**Figure 5:**
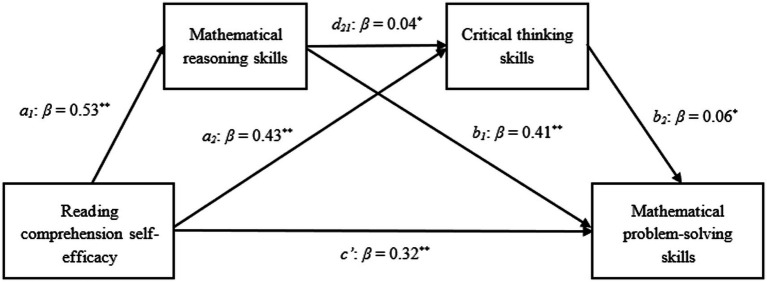
The sequential mediating roles of MRS and CTS in the association between RCSE and MPSS. *p < 0.05, **p < 0.01

The findings in Table 5 indicate that primary school students’ RCSE was positively and significantly associated with their MPSS (path *c*; *β* = 0.52, SE = 0.04, *p* < 0.01). Subsequently, the indirect effect of students’ RCSE on their MPSS was examined through the sequential mediating roles of mathematical reasoning and CTS. The findings indicated that mathematical reasoning and CTS served as sequential mediators in the association between students’ RCSE and MPSS (path *a_1_db_2_*; *β* = 0.01; 95% CI [0.00, 0.02], SE = 0.01). RCSE was significantly associated with MRS, the first mediator (path *a_1_*; *β* = 0.53, SE = 0.07, *p* < 0.01). In addition, MRS was significantly associated with CTS, the second mediator (path d*_21_*; *β* = 0.04, SE = 0.02, *p* < 0.05). This finding suggests that mathematical reasoning is positively associated with critical thinking within the sequential mediation model. Furthermore, CTS was significantly associated with students’ mathematical problem-solving skills (path *b_2_*; *β* = 0.41, SE = 0.06, *p* < 0.01). When the mediators were included in the model, RCSE remained significantly associated with MPSS (path *c′; β* = 0.32, SE = 0.05, *p* < 0.01). That is, RCSE remained a significant predictor of MPSS even after accounting for mathematical reasoning and CTS. This result indicates that the sequential mediating roles of mathematical reasoning and CTS are significant. These findings suggest partial mediation in the association between RCSE and MPSS. Furthermore, the fully standardized indirect effect of students’ RCSE on their MPSS was also significant (*K*^2^ = 0.03). These findings support the fifth hypothesis, indicating a significant sequential mediating pattern (see Figure 5). When examining the findings of the second series of mediating effects presented in Table 5, it was determined that critical thinking and MRS did not significantly mediate the association between RCSE and MPSS (path *a₂db₁*; *β* = 0.01; 95% CI [−0.00, 0.02], SE = 0.00).

## Discussion

This research aims to investigate the variables that are associated with the MPSS of primary school children, thereby corroborating existing literature and broadening the scope of prior investigations. Another important design feature of the present study is the inclusion of fourth-grade students from 14 provinces across Türkiye’s seven geographical regions. This geographical coverage increases the contextual relevance of the findings for Turkish primary school settings by reducing the likelihood that the observed associations are specific to a single province or region. Therefore, the relationships among RCSE, MRS, CTS, and MPSS can be discussed with reference to a broader range of Turkish primary school educational contexts. However, this should be understood as contextual relevance rather than unrestricted statistical generalization, given the study’s cross-sectional, correlational design. The study’s main finding is that mathematical reasoning and CTS significantly statistically account for part of the association between RCSE and MPSS. Building on Piagetian underpinnings, current research on mathematical cognition emphasizes the importance of relational thinking in relation to mathematical cognition. Relational reasoning entails seeing and cognitively manipulating relationships between entities, which is associated with pattern identification, analogical thinking, and the ability to generalize from specific cases to abstract principles (Richland et al., 2006). RCSE is a motivational and resource management variable that may be related to how effectively limited cognitive capacity is employed in problem-solving phases. Existing research indicates that RCSE is associated with mathematics achievement (problem solving and reasoning) (Nicolaidou and Philippou, 2003; Carroll and Fox, 2017). It suggests how RCSE may be associated with behavioral strategies and cognitive processes related to mathematical achievement (Kenney-Benson et al., 2006; Wright, 2010). However, RCSE may show a weaker association with solely computational mathematics (which does not involve reading) and a stronger association with mathematical problem-solving (which does). Numerous investigations have also reported findings consistent with this domain-specific permeability (Björn et al., 2016; Boctot et al., 2025; Boonen et al., 2016; Can, 2020; Frank and John, 1995; Hadianto et al., 2021; Lee and Kung, 2018; Nicolas and Emata, 2018; Shell et al., 1995; Supontawanit and Lertlit, 2021). In addition to self-efficacy in reading comprehension, metacognitive awareness is closely associated with MPSS. A study conducted by Bayraktar and Sümen (2024) found that self-efficacy in reading comprehension and metacognitive awareness statistically accounted for 16% of the variance in problem-solving success.

The MPSS of primary school children was found to be positively and significantly correlated with their RCSE, mathematical reasoning, and CTS. Reading comprehension self-efficacy appears to be associated with the quantity and quality of problem-solving experiences that may be related to schema-related mathematical thinking (Chi et al., 1981; Erdem, 2016; Kanwal et al., 2024; Kocadağ, 2019; Nahdi et al., 2023; Nahdiyah et al., 2020). Furthermore, research reporting associations between mathematical reasoning and reading comprehension self-efficacy suggests a possible association between self-efficacy and achievement (Fuchs et al., 2015; Honicke and Broadbent, 2016; Peng et al., 2020; Rojas and Benakli, 2020). On the other hand, studies (Kurnaz, 2018) that observed a positive relationship between problem-solving scores and reasoning scores are consistent with the results of our research.

This study also concluded that critical thinking significantly statistically accounts for part of the association between RCSE and MPSS. Regarding the indirect association involving critical thinking, it has been determined that critical thinking is associated with stronger ability of individuals to analyze a problem, evaluate different solutions, and make more appropriate decisions (Firdaus et al., 2015; Haeruman et al., 2024; Halpern, 2003; Harjo et al., 2019). RCSE may be associated with students’ engagement in the comprehension demands of mathematical word problems and with critical thinking in mathematical contexts (Basmaz and Kutlu, 2021; Sweller, 1988; Usta, 2021; Verschaffel et al., 2000; Yıldırım and Demir, 2024). A study by Vilenius-Tuohimaa et al. (2008) found that poor reading skills were negatively associated with MPSS. Students’ confidence in their cognitive abilities may be related to their willingness to engage in demanding critical analysis (Phan, 2010; Huitt, 1998). Critical thinking and problem-solving abilities are positively associated with one another (Tümkaya et al., 2009). Once more, Ocak et al. (2023) found that CTS was a significant statistical predictor of problem-solving abilities and appeared to be associated with in MPS processes. RCSE was positively associated with critical thinking, which is consistent with the present findings. Although mathematical reasoning and critical thinking were positively associated, they represent conceptually distinct constructs. Mathematical reasoning primarily involves identifying, representing, and manipulating mathematical relationships within domain-specific contexts, whereas critical thinking encompasses the evaluation, justification, and monitoring of alternative solutions and arguments across contexts. The present findings support the view that these constructs are related yet distinguishable cognitive processes that may contribute to mathematical problem-solving in complementary ways.

The study also determined that MRS statistically accounts for part of the association between RCSE and MPSS. This indirect association can be interpreted in relation to Baron and Kenny's (1986) definition of mediation, suggesting that self-efficacy beliefs may be associated with the initiation and sustained application of cognitive strategies, which in turn relate to problem-solving performance.

The study’s conclusion is that there is a statistically significant serial indirect association between RCSE and MPSS involving mathematical reasoning and CTS. This suggests that the association between self-efficacy and problem-solving performance is statistically consistent with a model in which mathematical reasoning and critical thinking operate sequentially; however, the cross-sectional design does not permit conclusions regarding developmental or causal ordering. It is possible that high reading self-efficacy, in the absence of sufficient mathematical reasoning skills, may not be sufficient to be strongly associated with effective solution strategies, suggesting that self-efficacy alone may not account for problem-solving competence. The present findings are consistent with the view that reasoning was positioned before critical thinking in the tested model critical thinking in this context, given that critical evaluation presupposes some representational content to assess — though the cross-sectional design of this study precludes conclusions about temporal ordering. This sequential pattern may be interpreted as a possible configuration of associations in problem-solving; however, given the correlational nature of the data, this interpretation should be regarded as speculative. The data are consistent with a model in which reasoning-based representations are positioned before critical evaluation, though directionality cannot be established from the current cross-sectional design. Theoretical accounts of cognitive development suggest that generative processes may precede evaluative processes (Kuhn, 1999; Moshman and Geil, 1998). Within the tested model, this sequential pattern is theoretically consistent with critical thinking being associated with evaluative aspects of problem solving; however, this interpretation remains tentative given the cross-sectional nature of the data.

When the serial indirect association was examined, it was observed that the model was not statistically significant when critical thinking was used as the first variable. However, the model was significant when mathematical reasoning was taken as the first variable. The difference in model significance stemming from variable ordering appears to differ from some findings in the literature. Both mathematical reasoning and critical thinking are viewed as parallel or mutually reinforcing skills in the problem-solving process. However, the present findings suggest that mathematical reasoning may be more strongly associated with critical thinking in the tested sequence within the context of this model. This is consistent with the view (Lester, 2007) that making sense of mathematical relationships may be theoretically positioned before the evaluation of solution strategies, though the present design does not permit causal or temporal conclusions. In this respect, the findings highlight a potentially stronger statistical association of mathematical reasoning compared to critical thinking within the sequential framework tested in this study. This approach is consistent with the view that before solving a mathematical problem, students may first make sense of the relationships and numbers in the problem (reasoning) and then critically evaluate different solution methods; which is aligned with studies suggesting that reasoning may serve as an important be closely associated with problem solving (Anggoro et al., 2023; Çelik and Özdemir, 2020). In Piaget’s staged development model, abstract and higher-order thinking skills (such as critical thinking) are theorized to be developmentally related to more concrete and logical operational thinking skills (such as mathematical reasoning). Primary school students are also considered to be in the process of transitioning from the concrete to the abstract operational stage. Furthermore, understanding the numerical relationships, patterns, and logical structures in a problem may be a more accessible skill for students in this age group. This corresponds to the “Understanding the Problem” and “Making a Plan” stages of Pólya’s (1945) problem-solving steps. After reading the problem, students may attempt to understand it within a mathematical framework- a process that, according to the present findings, may be associated with critical thinking in the tested model, though longitudinal designs would be needed to confirm this sequence (Lithner, 2008). CTS are more complex and cognitively demanding processes, such as questioning different aspects of the problem, comparing multiple solution strategies, and selecting the most effective. The “analytical intelligence,” discussed in Sternberg’s (1985) theory of triad intelligences, encompasses CTS. The findings suggest that the effective use of such higher-level cognitive processes may be associated with foundational reasoning skills. Accordingly, the results are consistent with a sequential pattern in which mathematical reasoning is associated with critical thinking. It is important to distinguish between statistical significance and practical significance when interpreting the sequential indirect pathway tested in this study. Although the sequential pathway involving RCSE, MRS, CTS, and MPSS was statistically significant, its practical magnitude was small. This indicates that the full sequential pathway accounts for only a limited portion of the association between reading comprehension self-efficacy and mathematical problem-solving skills. In comparison, the individual indirect pathways, particularly the pathway involving critical thinking, appear to have greater practical relevance. Therefore, the sequential pathway should not be interpreted as the dominant explanatory pattern in the model. Rather, the findings suggest that mathematical reasoning and critical thinking are both relevant to the association between RCSE and MPSS, while their individual indirect associations may be more practically meaningful than the full sequential pathway. Given the study’s cross-sectional design, this comparison should be interpreted as a difference in the strength of statistical associations rather than as evidence of causal or developmental ordering. On the other hand, some researchers, such as Sternberg (2010), argue that developing CTS becomes more pronounced in older age groups and with more complex cognitive tasks. Therefore, primary school students may initially develop the ability to establish mathematical relationships (reasoning), which may constitute a factor associated with critical thinking.

This finding can also guide classroom diagnostic assessment practices. The study shows that instead of interpreting students’ failure in mathematical problem-solving as a uniform difficulty, the approach can be structured to help distinguish whether the student’s difficulty stems from a linguistic barrier related to reading comprehension self-efficacy, a deficiency in mathematical reasoning, or a weakness in evaluating solution strategies. This effort is consistent with Newman Error Analysis (White, 2010), which helps teachers identify where students are experiencing difficulties by breaking down errors in mathematical word problems into reading, comprehension, transformation, processing, and encoding stages. The fact that a student performs successfully when the problem is presented in a restructured language (read aloud, visual support, etc.), even though they cannot solve the word problem independently, suggests that the difficulty may be more related to linguistic processing processes or reading comprehension self-efficacy. Conversely, the inability of a student to appropriately represent the quantitative relationships in the problem text, identify the relevant mathematical operations, or transform the given information into a coherent mathematical structure, even though they understand the problem text, suggests that the difficulty may be related to mathematical reasoning processes. The student’s inability to compare alternative solution strategies, evaluate the appropriateness of the outcome, and justify why their chosen strategy is more functional, despite constructing a mathematically plausible solution, suggests that the difficulty may be related to critical thinking and strategy evaluation processes. This situation demonstrates the importance of diagnostic assessment tasks and aligns with evidence-based assessment design, which emphasizes that these tasks should be developed to reveal observable evidence of specific student competencies rather than simply labeling answers as right or wrong (Mislevy, 2004; Newton et al., 2021). More specifically, this diagnostic process can be structured around three interrelated teacher competencies. First, teachers should be able to propose or select diagnostic tasks that reveal distinct sources of difficulty, consistent with the view that assessment tasks should align with assumptions about student cognition and yield interpretable evidence of students’ competencies (Chapman, 2023). At this stage, the same mathematical problem could be presented in its original written form with oral reading or visual aids, along with an additional question requiring students to justify or compare alternative solution strategies. Second, teachers should be able to assess the cognitive and emotional evidence contained in students’ responses. Difficulties in identifying given and requested information may indicate problems with reading comprehension self-efficacy or linguistic processing; difficulties in representing quantitative relationships may indicate limitations in mathematical reasoning; and difficulties in justifying, comparing, or evaluating solution strategies may indicate weaknesses in critical thinking. This finding; This is consistent with research that emphasizes teachers’ diagnostic competence as the process of recognizing, interpreting, and using evidence about students’ ways of thinking (Crismond and Adams, 2012; Herppich et al., 2018; Loibl et al., 2020). Third, teachers should use these diagnostic inferences to decide on specific instructional interventions, such as text comprehension aids, reasoning-focused representations, or strategy assessment discussions. In mathematics education, this type of diagnostic assessment is important because it can provide detailed information about students’ misunderstandings, reasoning processes, and instructional decision-making (Ketterlin-Geller and Yovanoff, 2009). Therefore, diagnostic assessment competence requires teachers to evaluate not only students’ cognitive knowledge components but also their confidence, perseverance, attitudes, and values toward mathematical problem-solving. Within this framework, teachers can identify and use short diagnostic task sequences that differ in linguistic, reasoning, and strategy-evaluation demands to determine students’ specific support needs in problem-solving. Since the current research has a cross-sectional, correlational design, such diagnostic interpretations should be considered pedagogical assumptions rather than causal explanations.

### Limitations

Several limitations should be acknowledged in interpreting the findings of this study. First, the research design was cross-sectional, which restricts the ability to infer causal relationships among RCSE, MRS, CTS, and MPSS. Longitudinal or experimental designs would allow for a more precise examination of developmental and causal dynamics between these variables. Second, the data were collected from a single educational context and limited age group of primary school students; therefore, the generalizability of the results to different cultural or educational settings remains constrained. Third, the study relied on self-report and performance-based instruments, which may be subject to social desirability bias and individual differences in interpretation. An additional limitation concerns the use of observed composite scores in the mediation analyses. Although the factorial validity of the instruments was established through CFA, the PROCESS analyses did not explicitly account for measurement error. Consequently, the reported path coefficients may represent conservative estimates of the associations among the constructs. Future studies may benefit from testing the proposed mediation model using latent-variable structural equation modeling approaches. Finally, unmeasured variables such as metacognitive awareness, motivation toward mathematics, or language proficiency could have influenced the observed relationships, suggesting the need for more comprehensive models in future research. Accordingly, the serial mediation model tested in this study should be interpreted as a theoretically informed pattern of statistical associations rather than evidence of developmental, temporal, or causal processes.

In this study, gender was not examined as a moderating variable. Given the reported gender differences in reading comprehension self-efficacy and mathematics performance (Kenney-Benson et al., 2006), future research should evaluate whether the sequential mediation model works equally for girls and boys using multigroup structural equation modeling (SEM) or moderated mediation analyses.

The limited variance explained for MRS (*R*^2^ = 0.11) suggests that RCSE should be interpreted as a contributing factor to mathematical reasoning rather than a comprehensive explanatory factor. This finding is consistent with the view that mathematical reasoning and performance are shaped by multiple cognitive and emotional processes. Mathematical tasks require the temporary storage, processing, and coordination of numerical, verbal, and visuospatial information in problem-solving (Baddeley, 2003; Peng et al., 2020). On the other hand, math anxiety can hinder mathematical performance by depleting limited working memory resources and disrupting efficient cognitive processing during mathematical tasks (Ashcraft and Kirk, 2001; Barroso et al., 2021). Individuals’ prior mathematical knowledge may also be another explanatory factor for additional variance. Prior mathematical knowledge has been shown to predict subsequent mathematical achievement, as well as general cognitive skills such as working memory and fluid intelligence (Stelzer et al., 2024). Therefore, future studies should test more comprehensive models that include RCSE along with working memory capacity, math anxiety, prior mathematical knowledge, and other relevant cognitive and motivational variables.

### Future research

Future studies should expand upon these findings by employing longitudinal or intervention-based methodologies to examine how RCSE, MRS, and CTS co-develop over time and interact dynamically in relation to mathematical problem-solving performance. Experimental studies designed to enhance RCSE or mathematical reasoning could clarify the directionality of effects suggested by the present model. In addition, incorporating qualitative approaches, such as classroom observations or think-aloud protocols, may provide deeper insights into the cognitive and motivational mechanisms underlying these constructs. Future research should also examine these relationships in diverse educational systems, age groups, and cultural contexts to assess the model’s cross-cultural validity. Moreover, integrating variables such as metacognitive regulation, self-regulated learning, and mathematics anxiety could enrich the theoretical framework and improve explanatory power. Finally, exploring the role of AI-supported learning environments and digital tools in fostering reading comprehension and reasoning skills could open promising pathways for innovative instructional practices.

## Conclusion

This study contributes to the growing body of literature linking literacy-related self-efficacy to mathematical performance by suggesting that mathematical reasoning and critical thinking may sequentially mediate the relationship between RCSE and MPSS among primary school students. The results highlight that self-efficacy in reading comprehension was associated not only with comprehension of problem texts but also with reasoning processes that may serve as a basis for critical evaluation and problem-solving performance. Mathematical reasoning appeared to function as a foundational cognitive process, while critical thinking was associated with an evaluative role in relation to reasoning outcomes, though these functional distinctions remain tentative given the cross-sectional design. These findings are broadly consistent with theoretical perspectives such as Piagetian cognitive development theory and Pólya’s problem-solving model; however, the present cross-sectional data do not permit direct tests of developmental sequences or causal mechanisms. Overall, the study underscores the interdependence of reading comprehension self-efficacy, mathematical reasoning, and critical thinking in relation to mathematical problem-solving during the foundational years of schooling. These findings suggest that instructional design in mathematics education may benefit from addressing not only computational procedures but also the textual, representational, and evaluative demands of word problems. In practice, mathematics tasks may be designed to help students first identify relevant linguistic information in the problem text, then represent mathematical relationships, and finally compare and evaluate alternative solution strategies.

The findings also have implications for mathematics teachers’ professional competencies, particularly regarding diagnostic assessment. Teachers may need to distinguish whether a student’s difficulty in problem-solving is primarily due to low confidence in understanding the problem text, insufficient mathematical reasoning, or limited ability to evaluate the appropriateness of solution strategies. For this purpose, teachers can use targeted diagnostic questions, think-aloud activities, short written explanations, and step-by-step problem-solving rubrics. For example, questions such as “What information is given in the problem?,” “What mathematical relationship do you see?,” and “Why did you choose this solution strategy?” may help teachers identify whether the difficulty is related to reading comprehension self-efficacy, reasoning, or critical evaluation. Such diagnostic practices may support more differentiated instructional responses, such as text-comprehension scaffolds, reasoning-focused representations, or strategy-evaluation discussions, depending on the source of students’ difficulties.

Given the cross-sectional nature of the present study, these implications should be interpreted as pedagogical suggestions derived from observed statistical associations rather than evidence of causal effects. Future longitudinal and intervention-based studies are needed to examine whether instructional practices that integrate reading comprehension, mathematical reasoning, and critical thinking are associated with improvements in mathematical problem-solving over time.

## Data Availability

The original contributions presented in the study are included in the article/Supplementary material, further inquiries can be directed to the corresponding author.
