# Supplementary material for: Reading comprehension self-efficacy and mathematical problem-solving in primary education: a serial mediation model through mathematical reasoning and critical thinking
Source: Front Psychol. 2026 Jul 7;17:1841685. doi: 10.3389/fpsyg.2026.1841685 (PMC13385453; doi:10.3389/fpsyg.2026.1841685)
Supplement: Supplementary file 1 [file Data_Sheet_1.docx]

A latent-variable structural equation model corresponding to the Hayes PROCESS Model 6 structure was established to test the serial mediating roles of mathematical reasoning and critical thinking in the effect of reading comprehension self-efficacy on problem solving. In the model, reading comprehension self-efficacy was defined as the independent latent variable, mathematical reasoning as the first mediator latent variable, critical thinking as the second mediator latent variable, and problem solving as the dependent latent variable.

The analyses were conducted in R using the lavaan package. Considering the ordinal categorical structure of the item-level indicators, the WLSMV estimator and delta parameterization were used. The critical thinking and problem solving constructs were modeled as second-order latent variables based on their theoretical subdimensions. Direct effects, specific indirect effects, the serial indirect effect, the total indirect effect, and the total effect were estimated together in the model.

**Table 1.**

*Fit Indices of the Latent-Variable SEM Model*

| Fit index | Value |
| --- | --- |
| χ², scaled | 3673.291 |
| df | 2836 |
| p | < .001 |
| CFI, scaled | .950 |
| TLI, scaled | .948 |
| RMSEA, scaled | .024 |
| SRMR | .060 |
| Robust CFI | .667 |
| Robust TLI | .656 |
| Robust RMSEA | .073 |

*Note. The model was estimated with the WLSMV estimator and delta parameterization. CFI = Comparative Fit Index; TLI = Tucker-Lewis Index; RMSEA = Root Mean Square Error of Approximation; SRMR = Standardized Root Mean Square Residual. The scaled fit indices indicate acceptable/good model fit. However, because the robust CFI and TLI values are low, model fit should be interpreted with caution.*

The scaled fit indices of the model indicated an acceptable level of fit, χ²(2836) = 3673.291, p < .001, CFI = .950, TLI = .948, RMSEA = .024, and SRMR = .060. However, the robust fit indices were found to be lower, robust CFI = .667, robust TLI = .656, and robust RMSEA = .073. In addition, a warning was received indicating that some latent variable residual variances in the model were estimated as negative. The inspections showed that this situation was particularly related to the curiosity dimension of critical thinking and the metacognition dimension of problem solving. Therefore, although the structural paths are interpretable, the latent SEM results should be evaluated cautiously in terms of the measurement model.

**Table 2.**

*Standardized Factor Loadings for the Measurement Model*

| Latent variable | Indicator | Standardized factor loading |
| --- | --- | --- |
| Reading comprehension self-efficacy | oa1 | .742 |
| Reading comprehension self-efficacy | oa2 | .690 |
| Reading comprehension self-efficacy | oa3 | .603 |
| Reading comprehension self-efficacy | oa4 | .563 |
| Reading comprehension self-efficacy | oa5 | .592 |
| Reading comprehension self-efficacy | oa6 | .502 |
| Reading comprehension self-efficacy | oa7 | .602 |
| Reading comprehension self-efficacy | oa8 | .583 |
| Reading comprehension self-efficacy | oa9 | .675 |
| Reading comprehension self-efficacy | oa10 | .614 |
| Reading comprehension self-efficacy | oa11 | .564 |
| Reading comprehension self-efficacy | oa12 | .542 |
| Reading comprehension self-efficacy | oa13 | .628 |
| Reading comprehension self-efficacy | oa14 | .647 |
| Reading comprehension self-efficacy | oa15 | .594 |
| Reading comprehension self-efficacy | oa16 | .675 |
| Reading comprehension self-efficacy | oa17 | .540 |
| Reading comprehension self-efficacy | oa18 | .607 |
| Reading comprehension self-efficacy | oa19 | .594 |
| Reading comprehension self-efficacy | oa20 | .626 |
| Reading comprehension self-efficacy | oa21 | .546 |
| Reading comprehension self-efficacy | oa22 | .546 |
| Reading comprehension self-efficacy | oa23 | .609 |
| Reading comprehension self-efficacy | oa24 | .594 |
| Reading comprehension self-efficacy | oa25 | .660 |
| Reading comprehension self-efficacy | oa26 | .664 |
| Reading comprehension self-efficacy | oa27 | .617 |
| Reading comprehension self-efficacy | oa28 | .616 |
| Reading comprehension self-efficacy | oa29 | .591 |
| Mathematical reasoning | m1 | .547 |
| Mathematical reasoning | m2 | .679 |
| Mathematical reasoning | m3 | .658 |
| Mathematical reasoning | m4 | .592 |
| Mathematical reasoning | m5 | .722 |
| Mathematical reasoning | m6 | .732 |
| Mathematical reasoning | m7 | .589 |
| Mathematical reasoning | m8 | .645 |
| Mathematical reasoning | m9 | .583 |
| Mathematical reasoning | m10 | .525 |
| Mathematical reasoning | m11 | .634 |
| Mathematical reasoning | m12 | .529 |
| Mathematical reasoning | m13 | .699 |
| Mathematical reasoning | m14 | .640 |
| Critical thinking: Skepticism | ed11 | .707 |
| Critical thinking: Skepticism | ed18 | .655 |
| Critical thinking: Skepticism | ed20 | .729 |
| Critical thinking: Skepticism | ed25 | .640 |
| Critical thinking: Curiosity | ed3 | .612 |
| Critical thinking: Curiosity | ed7 | .588 |
| Critical thinking: Curiosity | ed12 | .659 |
| Critical thinking: Curiosity | ed23 | .580 |
| Critical thinking: Curiosity | ed26 | .623 |
| Critical thinking: Open-mindedness | ed5 | .558 |
| Critical thinking: Open-mindedness | ed13 | .549 |
| Critical thinking: Open-mindedness | ed14 | .708 |
| Critical thinking: Open-mindedness | ed21 | .728 |
| Critical thinking: Open-mindedness | ed32 | .631 |
| Critical thinking: Bias | ed15_ters | .625 |
| Critical thinking: Bias | ed16_ters | .877 |
| Critical thinking: Bias | ed24_ters | .635 |
| Critical thinking: Bias | ed28_ters | .702 |
| Problem solving: Self-assessment | pc1 | .545 |
| Problem solving: Self-assessment | pc9 | .528 |
| Problem solving: Self-assessment | pc8 | .705 |
| Problem solving: Self-assessment | pc10 | .539 |
| Problem solving: Self-assessment | pc12 | .751 |
| Problem solving: Self-assessment | pc2 | .640 |
| Problem solving: Self-assessment | pc11 | .642 |
| Problem solving: Self-assessment | pc3 | .594 |
| Problem-solving knowledge | pc15 | .571 |
| Problem-solving knowledge | pc16 | .591 |
| Problem-solving knowledge | pc14 | .661 |
| Problem-solving knowledge | pc13 | .655 |
| Problem solving: Metacognition | pc4 | .581 |
| Problem solving: Metacognition | pc6 | .552 |
| Problem solving: Metacognition | pc5 | .603 |
| Problem solving: Metacognition | pc7 | .656 |

*Note. All values are standardized factor loadings obtained with the WLSMV estimator and delta parameterization. The critical thinking and problem solving constructs were modeled as second-order latent variables.*

**Table 3.**

*Second-Order Factor Loadings*

| Second-order latent variable | First-order factor | Standardized factor loading |
| --- | --- | --- |
| Critical thinking | Skepticism | .927 |
| Critical thinking | Curiosity | 1.012 |
| Critical thinking | Open-mindedness | .935 |
| Critical thinking | Bias | -.372 |
| Problem solving | Self-assessment | .893 |
| Problem solving | Problem-solving knowledge | .973 |
| Problem solving | Metacognition | 1.045 |

*Note. Standardized factor loadings above 1.00 were obtained for the first-order factors of curiosity and metacognition. When evaluated together with the negative residual variance warning produced by lavaan, this situation indicates a Heywood-type estimation problem. Therefore, the second-order measurement model should be interpreted with caution.*

**Table 4.**

*Structural Path Coefficients for the Latent Serial Mediation Model*

| Path | **B** | SE | **z** | **p** | 95% CI | **β** |
| --- | --- | --- | --- | --- | --- | --- |
| Reading comprehension self-efficacy -> Mathematical reasoning | 0.293 | 0.045 | 6.473 | < .001 | [0.204, 0.382] | .397 |
| Reading comprehension self-efficacy -> Critical thinking | 0.569 | 0.054 | 10.478 | < .001 | [0.463, 0.675] | .644 |
| Mathematical reasoning -> Critical thinking | 0.168 | 0.059 | 2.864 | .004 | [0.053, 0.283] | .140 |
| Reading comprehension self-efficacy -> Problem solving | 0.178 | 0.057 | 3.140 | .002 | [0.067, 0.289] | .272 |
| Mathematical reasoning -> Problem solving | 0.032 | 0.045 | 0.713 | .476 | [-0.056, 0.121] | .036 |
| Critical thinking -> Problem solving | 0.326 | 0.062 | 5.216 | < .001 | [0.203, 0.448] | .439 |

*Note. B = unstandardized coefficient; SE = standard error; β = standardized coefficient; CI = confidence interval.*

According to the structural model results, reading comprehension self-efficacy positively and significantly predicted mathematical reasoning, β = .397, p < .001. Reading comprehension self-efficacy also positively and significantly predicted critical thinking, β = .644, p < .001. Although the effect of mathematical reasoning on critical thinking was small, it was significant, β = .140, p = .004. In the prediction of problem solving, reading comprehension self-efficacy, β = .272, p = .002, and critical thinking, β = .439, p < .001, were identified as significant predictors. In contrast, the direct effect of mathematical reasoning on problem solving was not significant, β = .036, p = .476.

**Table 5.**

*Direct, Indirect, Serial Indirect, and Total Effects*

| Effect | **B** | SE | **z** | **p** | 95% CI | **β** |
| --- | --- | --- | --- | --- | --- | --- |
| OA -> MATH -> PC | 0.009 | 0.013 | 0.729 | .466 | [-0.016, 0.035] | .014 |
| OA -> ED -> PC | 0.185 | 0.038 | 4.921 | < .001 | [0.112, 0.259] | .283 |
| OA -> MATH -> ED -> PC | 0.016 | 0.006 | 2.629 | .009 | [0.004, 0.028] | .024 |
| Total indirect effect | 0.211 | 0.041 | 5.101 | < .001 | [0.130, 0.292] | .322 |
| Total effect | 0.389 | 0.048 | 8.128 | < .001 | [0.295, 0.483] | .593 |

*Note. The specific indirect effect through mathematical reasoning is not significant. The indirect effect through critical thinking and the serial indirect effect through mathematical reasoning and critical thinking are significant.*

When the indirect effects were examined, the indirect effect of reading comprehension self-efficacy on problem solving through mathematical reasoning was found to be non-significant, β = .014, p = .466. In contrast, the indirect effect through critical thinking was significant, β = .283, p < .001. In addition, the serial indirect effect in the form of reading comprehension self-efficacy -> mathematical reasoning -> critical thinking -> problem solving was also found to be significant, β = .024, p = .009. The total indirect effect, β = .322, p < .001, and the total effect, β = .593, p < .001, were significant. These findings show that reading comprehension self-efficacy is related to problem solving both directly and indirectly, especially through the serial path involving critical thinking and mathematical reasoning together with critical thinking.

**Table 6.**

*Explained Variance Values for Endogenous Latent Variables*

| Endogenous latent variable | **R²** |
| --- | --- |
| Mathematical reasoning | .158 |
| Critical thinking | .506 |
| Problem solving | .456 |

*Note. R² values indicate the proportion of explained variance in the relevant endogenous latent variable.*

**Methodological Positioning That Can Be Added to the Discussion Section**

Latent-variable SEM has the advantage of explicitly incorporating measurement error into the model. However, the full item-level latent model in this study is statistically quite complex and produced some estimation irregularities. Therefore, the latent SEM analysis was considered not as an unconditional alternative to the PROCESS Model 6 analysis but as an additional robustness analysis. The PROCESS results and latent SEM results largely point to the same theoretical conclusion: the indirect path through critical thinking is significant, the serial indirect path through mathematical reasoning and critical thinking is significant, and the indirect path in which mathematical reasoning alone serves as the mediator is not significant in the latent model. This pattern of findings supports the robustness of the main theoretical interpretation and also shows that the limitations of both analytical approaches should be carefully considered.
